# A Practical Treatise on Inflammation, Ulceration, and Induration of the Neck of the Uterus: With Remarks on the Value of Leucorrhœa and Prolapsus Uteri as Symptoms of Uterine Disease

**Published:** 1846-01

**Authors:** 


### ART. XIV.

*A practical Treatise on Inflammation, Ulceration, and Induration of the Neck of the Uterus: with Remarks on the value of Leucorrhœa and Prolapsus Uteri as Symptoms of Uterine Disease.* By J. H. BENNET, M.D. &c. *London,* 1845. 8vo. pp. 212.

IN the study of uterine diseases we in England labour under disadvantages, which the French accoucheurs, particularly in hospital practice, are exempt from. He would be, indeed, a hardy pioneer, who would undertake to face the storm of outraged feelings which the full adoption of the French system of exploration of the os uteri, in either public or private practice in England, would excite. Even to the mind of an *English surgeon* there is something revolting in the nonchalant manner of exposing females in the Parisian wards. This feeling, if it is found among the members of the medical profession in this country, is of course reciprocated a hundredfold by the public. It may be that it is a weakness, a want of philosophy; whatever it may spring from, so it is, and, in consequence, our neighbours enter this path of inquiry with advantages which we possess not; and, as long as this is the case, we must yield to them, in some degree, the palm of practical acquaintance with those facts which the use of the speculum alone can reveal.

Not that the facts so discoverable have, in our estimation, the weight which some attach to them. The use of the speculum in French practice is so universal that the practitioners of that country are apt to despise all other means of investigation except as subsidiary to this; and therefore naturally give the most prominent and constant place to that which really might often be dispensed with. But we are happy to find that the use of this important means of investigation is increasingly adopted in England; and though we cannot hope or desire to see our national character so modified, as to find forty females at a séance submitting to a procedure, which must outrage all feelings of delicacy or decorum, yet, with proper precautions, and with all possible regard to decency, we do fully believe that this most valuable means of diagnosis may be practised both in public hospitals and in private practice.

We do not hesitate to join fully in the assertion of the intelligent author of the little book before us, that we have never found insuperable obstacles to the use of the speculum, and we cannot but attribute the comparatively rare employment of it more to the want of will in the surgeon than in the patient. We would strongly impress upon our surgical brethren that the value of the speculum consists, not more in its facilities for diagnosis, than in the *application of remedies,* and we do not hesitate to say, that all local remedies must be much more readily and perfectly applied with the speculum than without it.

But we must go farther, and say, that if the use of the speculum should not be more general than it is, the practice of the *touch,* as being more ready of application, and less likely to excite a feeling of opposition in the patient, should in all doubtful cases be considered indispensable. We are well aware that this practice, particularly in the country, is lamentably neglected. Patients are allowed to go on, week after week, month after month, to sink into an ill-understood but confirmed state of disease, and to death, without the least proposal being made by the surgeon to examine locally the nature and character of the affection. The accuracy which may be attained by practice in the detection of shades of disease by the finger is quite inconceivable by those who have only rarely practised it; and we hold it to be the bounden duty of every practitioner; who is in the habit of treating the diseases of females, to neglect no opportunity of examination of the os uteri by the touch, in order that he may acquire and perfect that discriminating sense of which the finger is capable.

We have been led to preface our observations upon Dr. Bennet's treatise with these remarks upon the means of investigating the diseases of the os uteri, from the conviction that a stimulus is greatly needed to induce practitioners of one of the most important branches of the healing art, both to make the most use of that means of investigation which they possess, and to take every opportunity of completing its value by the use of the speculum.

The opportunities which our author possessed of investigating uterine disease in Paris were considerable, and he has certainly made the most of them: with the results he makes us acquainted in his book; of which we feel it but fair to speak in terms of considerable praise, as the produce of much industry and accuracy of observation. But we could have dispensed with much unnecessary matter; and had the pruning and clipping been intrusted to our hands, before it was sent to the printers, we should have been inclined to have reduced its size to the really valuable portion of the matter. Notwithstanding, we augur well for the future success of the author, from the ability and industry shown in the present attempt, and wish him heartily well in the very important course of investigation which lies open to him.

The frequency of chronic inflammation of the neck of the uterus has often struck us as remarkable; and has in many instances been to us the clue to obscure diseases, the external manifestations of which are not at all to be trusted to. Cases of apparent spinal, lumbar, hypogastric, and even crural neuralgia, and a number of the anomalous cases of obstinate nervous diseases, may be traced, more or less directly, to a chronic state of inflammation of this part, with which the whole system most peculiarly sympathises.

We can further entirely sanction, by the result of our experience, the views of our author upon the frequency of the *symptoms* of prolapsus depending upon a chronic inflammation, and consequent hypertrophy of this portion of the female organ. We were long surprised at the amount of distress occasioned by symptoms of prolapsus uteri when on examination we have found but little descent, and the size of the *entire* uterus not much more than natural, though the cervix itself was hard and occasionally tender. We were at first so struck with this discovery, that we were inclined to doubt the reality of the relation. But observation has convinced us that the symptoms usually attributed to prolapsus are often produced simply by the engorgement and hypertrophy of this portion of the uterus; and that to relieve the symptoms they must be attacked through it, as the fons et origo mali. Nor do we consider that in these observations we are at all at variance with the authority of Dr. Ashwell, who, in his valuable work on the Diseases of Females, gives it as the result of his experience, that inflammation of the cervix is rare; as, from the statement of symptoms which he there gives, he is evidently speaking of acute inflammation of this part, whereas the cases which have fallen under our observation have all been of a chronic character, and probably never exhibited symptoms of an active nature. That the symptoms of prolapsus are by no means always proportionate in severity to the size attained by the uterus, as generally understood, or to the laxity of the vagina and round ligaments, we think few practitioners will maintain, who are in the habit of examining all their cases by the touch. That the existence of such a state of parts in women who have borne many children, and who have favoured the descent of the uterus by over exertion, will be productive of severe distress, we deny not; neither do we doubt that, in neglected cases of inflammation and hypertrophy of the cervix, the foundation of permanent enlargement and descent may be laid; but we do contend that there is a large number of cases which a superficial examination would set down as prolapsus, in which the sufferings are dependent upon other causes than those of prolapsus in general, and which will not be benefited by the ordinary appliances for prolapsus, but on the contrary injured by them. We are the rather induced to press this opinion from the conviction that the use of pessaries and mechanical supports, as they are called, is far too universal and too indiscriminate in these cases. This mode of palliation is all well enough where the prolapsus is produced by a very relaxed state of the vagina, and increase of size in the uterus, from frequent childbearing. But when this cause has not been in operation, and where the symptoms of prolapsus being present at the same time, the vagina being natural, and the body of the uterus natural, or nearly so, in size, and when the cervix is indurated and hypertrophied, the symptoms of prolapsus will be aggravated rather than diminished by the pessary, and the symptoms can be relieved only by the treatment being directed to the correction of the morbid state of the cervix. Yet we have frequently known these cases treated by the use of the pessary, and, as might be expected—assuming that the view we have just given of their cause and pathology be correct—with injury rather than benefit.

The observations of Dr. Bennet on the pathology and treatment of ulcerations of the cervix, in the uterus which has never been pregnant, are practical and valuable, and deserve to be well borne in mind, in forming a prognosis. But this class of cases is just that in which we should be inclined to dissent from his continual use of the speculum. In those slight cases which simply indicate a superficial ulceration, or a slight hypertrophy of the cervix, we can see no possible necessity for its use, or for putting the patient's feelings to the unpleasant trial of the proposal to employ it, and, we must add, the doctor to the unpleasantness of recommending it. Certainly, the examination by the touch is all that the necessity of the case requires for the diagnosis ; and the practised finger may here be safely relied on. At the same time we must allow, that if the application of the cautery, actual or potential, is required, the speculum is still essential for its application. But we further contend, that in these slight cases of which we speak, viz. those occurring in the uterus which has never been impregnated, other and milder forms of remedy will answer the end.

Dr. Bennet is sometimes visionary and too theoretical, as when he endeavours to prove the extreme frequency of laceration, followed by cicatrization and even ulceration, as the result of natural labours, (pp. 37-8.) We have so entire a confidence in that wonderful arrangement for the steady and natural, though vast change which takes place in the uterus, from the first deposit of the ovum to the last stage of the restoration of the maternal organs, that we have no hesitation in protesting against the adoption of an axiom founded on the idea that nature is unequal to the completion of her work.

We have seen no reason for believing that there is any laceration at all of the lining membrane or other structures of the uterus, in by far the majority of labours. But in fact we must warn Dr. Bennet's readers to bear continually in mind that his experience is principally drawn from hospital practice among the lowest class of prostitutes, and that he of course must be understood to be speaking always of that class : we do not therefore suppose that his observation of the opinion of M. Boys de Loury, that "ulceration of the cervix is common with pregnant women," is intended to have any further application than to the class in question, though the assertion is made without this qualification.

Upon these same grounds, as well as from the result of our own observations, we are disposed to demur to the conclusion that "the state of pregnancy predisposes to inflammation and ulceration of the cervix uteri." We here speak of the general run of cases, while it is evident that Dr. Bennet's conclusion can only be received as regarding the case of pregnancy in diseased syphilitic subjects. Had he qualified his assertion by this allowance, we had yielded direct assent, but then to the practitioner in midwifery the value of the dogma is materially diminished by this qualification.

When he enters into the province of cancerous ulcerations, our author tells us but little that is not universally received, and it would have been better modestly to have acknowledged this than to have assumed the character of guide through a *terra incognita* without any better pretensions to the character than many who have gone before. His diagnosis between simple, severe inflammatory ulceration and cancer fails entirely, as every attempt of the kind has failed, and must fail, by taking the character of an extreme case, and its features as those which are to be universally found, and in all stages of the disease. What we want as a diagnostic, is a universal and readily recognizable character which may indicate the genus in every locality and under every form and stage of growth. Dr. Hodgkin has gone further than any one else in accomplishing this most desirable object, and were the cystiform arrangement as readily recognizable during life as it is universal in its occurrence when examined after death, there would be little more to desire in the way of diagnosis, and cancer would be reduced to the same category under this head with all other ordinary diseases. This, however, is not the case, and we are still obliged to grope our way in the dark without the least spark of enlightenment from the past efforts of science. We are not without hopes that the forthcoming work of Dr. Walshe may remove some of our difficulties.

Dr. Bennet's cases of severe ulceration, both in the pregnant and unimpregnated state, are interesting; and his descriptions and cases of genuine chancre in the cervix uteri are well worth studying. (pp. 92 to 122.) On the subject of the treatment of obstinate ulceration and of hypertrophy of the cervix by deep cauterization, we shall only say that the plan is valuable if further trial gives the same amount of success which attends it, according to Dr. Bennet, in the Paris hospitals. We have not witnessed it, nor are we aware of its having been adopted in this country, but it may nevertheless be well worth the trial. In this practice our neighbours are bolder than we are, as indeed in surgery generally, which fact is strongly contrasted with what we should often call timid practice in medicine.

The deep cauterization is, according to Dr. Bennet, used with very general benefit and without any risk. In judicious and skilful hands it promises well in our estimation, and we should therefore strongly recommend those who are frequently in the habit of treating uterine diseases to turn their attention to a plan which gives great promise of benefiting permanently a most distressing class of cases.

We shall here append, in the form of extracts, the details of the principal local means of treatment recommended by Dr. Bennet, not only in these severer forms of disease, but in the slighter also: and we shall begin with the latter. Frenchified as the style will be found in these, they are far from unfavorable specimens of the manner in which Dr. Bennet sometimes travesties his mother-tongue. But his long residence in a foreign country is a better excuse than most delinquents in this way have to plead.

I. "*The treatment of inflammation and ulceration of the uterine neck* [*neck of the uterus*] *in women who have not borne children.*"

A. *Without ulceration.* "The remedies usually resorted to in general vaginitis are here indicated. The most efficacious is, undoubtedly, the solid nitrate of silver, lightly drawn over the inflamed cervix, and over that part of the superior parietes of the vagina which generally participates in the inflammation.....

"After the mucous surface has thus been slightly cauterized, emollient or slightly astringent injections (acetate of lead or sulphate of zinc) must be used three or four times a day; complete rest must be enjoined; sexual congress strictly forbidden; and the general health attended to.....

"On the parts being again examined, in the course of a week or ten days, the inflammation will generally be found to have subsided, unless it coexist with blennorrhagia, in which case it will prove much more difficult to eradicate, and requires the same treatment as in the other regions of the sexual organs. Sometimes, nevertheless, it will be found advisable or necessary to reapply the nitrate of silver to the mucous surface a second or a third time. This occurs more especially when the injections have been improperly performed, as is usually the case if the patient is not instructed as to the way in which they should be used. . .

"To obtain the full effect of the injection, the patient should recline on the side of a bed, or of a lounging chair, elevating the pelvis, so as for the vagina to form an inclined plane, of which the cervix is the most dependent point. The vagina thus retains the injected fluid, like a vase; it penetrates gradually into every part, and remaining in contact with the inflamed tissues for a few minutes, exercises a decided influence on them." (pp. 138-41.)

B. *With ulceration.* "The basis of the treatment of inflammatory *ulceration* of the cervix uteri is, the cauterization of the ulcerated surface. If the inflammation has not penetrated to the deep tissues of the cervix, and there is no general inflammatory hypertrophy of the organ, *superficial cauterization*, combined with emollient and astringent injections, rest both of the organ and of the system, and attention to the general health, is all the treatment that is required, and will generally effect a cure in from four to six or eight weeks.....

"The progress of the inflammation and ulceration is, generally speaking, at once arrested by the cauterization. The congestion and redness of the cervix diminish visibly, the granulations become smaller and healthier, and the purulent secretion assumes the character of laudable pus, if it has not presented it before. When the cauterization is suspended, the ulceration will, however, often remain stationary, even if other measures (injections, &c.) are resorted to; and if left entirely to itself it is nearly certain to relapse, however advanced the healing process may be.....

"The agents used for the cauterization of the ulcerated cervix are various. The principal are the nitrate of silver and the acid nitrate of mercury. The latter is exclusively employed by many Parisian practitioners, among whom I may name M. Emery. It is a dissolution [solution] of deuto-nitrate of mercury in nitric acid ....

"When recourse is had to the nitrate of silver, its application may be repeated every fourth or fifth day, whereas the acid nitrate of mercury should not be applied oftener than once a week. The eschar formed by the former, being much more superficial than that formed by the latter, falls much sooner. In either case, whenever cauterization is resorted to, whatever be the agent, the blood or mucus which covers the cervix must be wiped off before the caustic is applied, otherwise the greater part of its power is lost in coagulating these fluids. This can be done conveniently by means of a little lint or sponge, held in a pair of long forceps. Those which I use are thin, about ten inches in length, and made on purpose. The solid nitrate of silver, fixed in a long porte caustic, must be drawn two, three. or four times over the ulcerated surface, according to the effect wished to be obtained.

"In order to apply a fluid caustic, the following plan should be resorted to:— A small thin stick, about a foot in length, having been chosen, is formed into a brush, by inserting between its divided extremities a little wool, lint, or old linen, which is then fastened by a few turns of thread. These little brushes may be made *ex tempore*, and being of no value, can be thrown away when they have been used. The brush having been introduced into the acid, should be pressed against the rim of the bottle, in order that it may be merely moistened with the caustic, and then drawn over the ulcerated surface. A little water must then be injected into the speculum before withdrawing it, in order to absorb any superabundant acid.....

"In cauterizing the cervix, the speculum must be *firmly* applied to the parts, so as to protect the vagina from the action of the caustic." (pp. 144-50.)

II. "*The treatment of inflammation, ulceration, and induration of the uterine neck in women who have borne children.*

"In acute and recent cases of inflammation of the cervix in women who have borne children, the inflammatory nature of the hypertrophy is well marked by the intense redness and heat of the enlarged organ; but in chronic disease, these symptoms subside, leaving a passive hypertrophy, which appears to depend principally on a modification of the nutrition of the part. The redness is no longer vivid, and the heat is scarcely, if at all, greater than natural; indeed, the inflammation may be said to have ended in induration. Even in these cases—in which the disease has reached, as it were, one of its natural terminations—the ulceration of the surface of the organ often persists. This ulceration is kept up by the frictioning [!] of the hypertrophied organ against the parietes of the vagina.....

"The chronically enlarged cervix is extremely hard and resistant to the touch in its entire extent, and has not the latent elasticity which may still be observed in acute inflammatory hypertrophy. In either case, the size of the organ varies from that of a walnut to that of a large egg. The larger the volume, the more distressing are the symptoms produced by the prolapsus and retroversion of the cervix, so that the life of a woman who is labouring under hypertrophy of the cervix is often a burden to herself; she is never free from painful and disagreeable sensations.....

"As long as the uterus itself is acutely inflamed, no examination with the speculum should be attempted, as it would be very painful; but as soon as these inflammatory symptoms have subsided, it must be at once resorted to, as nothing will tend so much to allay the irritation of the uterine system as cauterization of the ulcerated surface. So far from cauterization in these cases, even when there is still acute inflammation of the cervix, and slight subacute inflammation of the uterus, endangering the life of the patient, as has been asserted, by exposing her to metritis, or metro-peritonitis, it invariably diminishes the inflammation both of the cervix and of the surrounding parts.....

"The measures which I recommended in inflammation and ulceration, unaccompanied by general induration, (viz., superficial cauterization, injections, rest, and a light diet,) will very often alone suffice both to cure the ulceration and to resolve the induration. . .

"This, however, only occurs when the nutrition of the engorged cervix has not been deeply modified by the subacute inflammation of which it has perhaps been the seat, for months or years Not unfrequently, especially in very chronic cases, the hypertrophy only diminishes to a certain extent under the above treatment, and even that very slowly, and then remains stationary, whether the ulceration heal or not.....

"Complete rest, which, in ulceration unaccompanied by induration, I stated to be only extremely desirable, is now indispensable. Indeed, if the patient can be prevailed upon to keep her bed for a few weeks, it is much the best plan. When walking, standing, or even sitting, the enlarged cervix drags down the uterus; whereas, when the patient is lying down, this does not occur If there is a good deal of hypogastric pain, large linseed poultices applied to the hypogastrium, and changed occasionally, will often give great relief. These poultices should be made thin, or otherwise their weight is painful. Tepid or cold hip-baths twice a day are useful adjuncts to the treatment.

"In these cases, cauterization of the ulcerated surface may generally be resorted to from the first, but the action of the nitrate of silver is too superficial, and the acid nitrate of mercury, or caustic potassa, should be preferred. Emollient or astringent injections should also be used When the inflammation is confined to the neck, emollient injections will suffice; if the vagina is also inflamed, astringent injections are indicated.....

"The treatment may be confined to these measures for two or three weeks, during which time the influence of the medication followed must be narrowly watched. If the ulceration becomes less angry looking, if the granulations assume a healthier appearance, and if the hypertrophy of the neck appears rapidly to decrease, the treatment may be continued, as it will probably prove quite sufficient to effect a complete cure. But if this is not the case, if the amelioration which at first takes place ceases, or if the ulceration appears inclined to heal without the induration giving way, other measures must be resorted to.

"The most efficacious is the application of leeches directly to the uterine neck itself. They are extremely useful agents in subduing deep-seated chronic inflammation in this region. The following is the easiest way to apply them: after introducing an ordinary conical metal* speculum, wipe off the mucus which covers the surface of the cervix with a little lint or sponge, and then place the leeches in the interior of the instrument. Over the external orifice of the speculum spread a piece of linen, which depress with the finger into the speculum. In the concavity thus formed, place some lint or cotton, and then, with the forceps, push the whole towards the uterine neck. The linen carries the leeches before it, and presses them against the os uteri. On pulling out the linen and the lint, with which the speculum was plugged, in the course of about ten minutes it will nearly always be found that all the leeches have taken. They generally fill well in this situation, and the flow of blood is often considerable after they have fallen.....

"In order to modify effectually an engorged cervix, which has resisted all other modes of treatment, the indurated organ must be deeply cauterized, either with the Vienna paste, the plan adopted by M. Gendrin, or by the actual cautery, that followed by M. Jobert, (de Lamballe.) The eschar which forms, in either case, is much deeper than that which is created when the fluid caustics are used. The inflammation which accompanies its separation is also much more intense, and generally propagates itself into the entire cervix. The result is, that not only is the hypertrophied cervix diminished by the extent of the eschar which separates, but that the healthy inflammation set up in the chronically indurated tissues gradually melts them, as it were; so that often, on its subsiding, the hypertrophied cervix has regained its natural size.....

"If the Vienna paste is employed, the following is the plan pursued by M. Gendrin, which I likewise follow. I must first, however, state that the Vienna paste, which is much used in France to produce deep eschars, is formed of equal parts of quicklime and hydrate of potassa, reduced to a fine powder, and intimately mixed. This powder should be prepared only when wanted, and kept in a glass-stoppered bottle. To be used, it is made into a paste with a few drops of alcohol, and the paste is then spread over the part to be destroyed ....

"When applied to the uterine cervix, a large and conical speculum must first be introduced, and the engorged cervix made to enter its orifice; or should the cervix be too voluminous, the speculum must be firmly pressed on the part which it is intended to cauterize, great care being taken not to inclose between the rim of the speculum and the cervix a fold of the vagina. About as much of the paste as would cover a fourpenny-piece, a line in thickness, must be placed on a triangular piece of diachylon plaster, one end of which is inserted lightly in the cleft extremity of a small stick The caustic paste is then carried by means of the stick to the cervix, and applied to the centre of the part comprised by the orifice of the speculum. With the long forceps, cotton is placed carefully all round the spot on which the caustic is applied, so as to completely protect the neighbouring parts; the stick having been withdrawn, the speculum is two-thirds filled with cotton or lint, which is firmly pressed against the uterine neck. The speculum is then extracted, the cotton which fills it being forcibly pushed back in the vagina with the forceps, as it is pulled away, so that the vagina remains thoroughly plugged. If all this is carefully done, it is impossible for the caustic to fuse, and to injure the parietes of the vagina. In about fifteen or twenty minutes, the cotton or lint must be gradually withdrawn by means of a bivalve speculum, and an eschar, of the size of a shilling, or rather larger, will be found where the caustic was applied. The vagina should then be washed out with a little tepid water, complete rest in bed enjoined, and emollient injections employed until the separation of the eschar, which takes place from the sixth to the eighth or tenth day." (pp. 138-78.)

We cannot part with our author without again expressing our high opinion of his little work, and recommending our brethren to possess themselves of it.

---

